# Malarial Keratitis

**Published:** 1881-12

**Authors:** F. C. Hotz

**Affiliations:** Chicago, Ophthalmic Surgeon, Illinois Charitable Eye and Ear Infirmary


### ARTICLE V.

MALARIAL KERATITIS. By F. C. HOTZ, M.D., Chicago, Ophthalmic Surgeon, Illinois Charitable Eye and Ear Infirmary.

Malarial diseases are so frequent, that it is singular how little we know of their effect upon the eyes. The most of text-books say nothing about it; only Macnamara * mentions malaria as a frequent and important cause of hyperæmia of the retina.

The fact is, we have but begun studying the very interesting subject of the influence which may be exercised upon the tissues of the eye by malarial poison or other febrific agents, after their introduction into the blood.

Directly bearing upon this question was the interesting communication which Prof. Horner, of Zurich, made to the German Ophthalmological Society in 1871.* He had observed the erup-

* Manual of Diseases of the Eye; 3rd Ed., 1876. p. 390.

* See Klein. Monatsbl. 1871.

tion of herpetic vesicles on the corneæ of thirty-one persons afflicted with pneumonia or other acute affections of the respiratory organs. Subsequent observers confirmed Horner's statement; but at the same time they found that other febrile diseases (intermittent fevers, acute affections of the intestines, etc.) may also produce herpes of the cornea. A very large collection of cases of this "herpes febrilis corneæ," as it is called, has recently be enpublished by Dr. Godo, of Paris.* Referring to malaria as a cause of corneal herpes, he says that in at least one-third of his cases he could trace the affection of the cornea to the influence of malarial fevers.

But the descriptions of the cases which Dr. Godo reports as instances of malarial herpes, differ in no essential feature from those of his herpes cases due to other causes. It would be impossible to make a differential diagnosis from the local manifestations which are described, alike for all cases, as groups of minute vesicles in the peripheric cornea, which subsequently are transformed into superficial irregular ulcers, healing readily under the appropriate local and general treatment.

It was left to the careful and patient observer, Dr. Chas. J. Kipp, of Newark, to discover the peculiar and characteristic features by which keratitis due to malaria can be readily recognized, and easily distinguished from similar corneal troubles. The description the doctor furnished to the American Ophthalmological Society in July, 1880, is so true and clear that I may be allowed to reproduce his own words:

"If the eye is examined shortly after the first symptoms of irritation are noticed by the patient, one or two or more slightly raised opaque lines, of varying length, will be found on different parts of the surface of the cornea. At the same time, some circumcorneal injection will be present. On the following day these opaque lines will have increased in length, whilst at the same time the middle portion of the opacity has been transformed into a shallow ulcer. Under favorable circumstances, no further increase in size takes place, the remaining opaque epithelium is thrown off, and reparation begins, to be completed only after several months. But not unfrequently the ulcer continues for

* De l'herpès fébrile de la cornée. *Recueil d'ophthalmologie*, Mars, Avril, Mai, 1880.

days, and even weeks, to grow slowly in length, and at the same time sends out club-shaped, slightly raised, grayish offshoots from its sides. In some of my cases the ulcer crept across the entire cornea, and in a few others, in which several ulcers appeared simultaneously on different parts of the cornea, the whole epithelial layer was eventually destroyed. As a rule, the ulcer shows no tendency to increase in depth, and its floor and edges are usually of a bluish-gray tint. The middle and inner layers of the cornea generally remain transparent throughout, but in neglected or maltreated cases, an extensive star-shaped opacity of a slightly yellowish-gray tint is sometimes developed in the inner layers of the central part of the cornea. An hypopyon is but rarely seen, even in the severest cases, and spontaneous perforation of the cornea did not occur in any of my cases. A decrease of the tension of the eye-ball, or a diminution of the sensibility of the cornea, could not be demonstrated in any of the cases. The development and growth of the ulcer was always accompanied by very severe pain in and around the eye, more especially along the course of the supra-orbital nerve, and by photophobia and lachrymation. The process of repair was commonly initiated by the extension of blood-vessels from the limbus conjunctivæ toward the ulcer, and was in all cases extremely slow, two to four months being generally required for its completion."

Should any one find it difficult to conceive from Dr. Kipp's description a distinct image of the peculiar form of these malarial ulcers of the cornea, let me assist his imagination by the aid of some familiar object in nature. Draw within the compass of the cornea the outlines of a small lanceolate leaf, with its stem at the margin and its free end in the center of the cornea ; have the central vein of this leaf run in a slightly zigzag course, and let the lateral veins be short. Now erase the outlines of the leaf, and the skeleton of the veins is a correct representation of the specific character of a malarial ulcer of the cornea. This form of the ulcer is as pathognomonic for malaria as the mucous patches in the mouth are for syphilis.

You will now appreciate the great importance of Dr. Kipp's discovery. It has revealed to us the signs by which we can recognize the constitutional cause of this form of corneal ulcers ; it has rendered our diagnosis independent from the statement of the patient. We can positively diagnosticate the malarial character of the ulcer, whether the patient admits or denies having had malarial fever. He who is not familiar with Dr. Kipp's specific symptoms, will probably regard the trouble as a simple keratitis or a superficial abrasion of the cornea, which he expects to relieve in a few days by local remedies ; but he will be sadly disappointed by finding that the ulcer has been daily increasing in length, and the pain, photophobia and other symptoms have not been subdued by the liberal use of atropia and the bandage.

Local treatment alone is insufficient. Starting from the margin of the cornea, the ulcer will extend longitudinally to and beyond the center of the cornea, and eventually also become broader by implicating the epithelium between the lateral offshoots. But give your patient quinine, and you will arrest the ulceration at any stage of its progress, and speedily relieve the patient of a very painful and troublesome affection. The amelioration supervenes upon the administration of quinine (two grains every two hours) so promptly that it must be attributed to the influence of this medicine. And when the surgeon has faithfully but vainly tried for a week or two to subdue the trouble by appropriate local treatment, the sudden change following directly upon the use of quinine is so suggestive and striking as to convince even the most skeptical Thomas.

A few cases may serve as illustrations to the foregoing remarks.

I. C. W., aged aged twenty-six, short-hand writer, consulted me on Jan. 24. Five days previously he had a chill followed by high fever at night. The next day his right eye became red and painful, and has been growing worse ever since.

I found the eye extremely sensitive even to moderate light; and every movement of the upper lid caused a sharp pain as if a gritty substance was cutting into the cornea. The tears were constantly running over the lower lid and the eye-ball showed considerable engorgement of the conjunctiva. In the cornea, near its upper margin, I discovered a minute linear abrasion of the epithelium. I prescribed the use of an ointment of atropine

(gr. ij to ℥j of vaseline), and dismissed the case. But on Jan. 29 the patient returned, stating that the treatment had given him no relief; that the pain had continued day and night, and that his sight had become very dim. The examination showed me that the patient was right, and had just grounds for his dissatisfaction. The inflammatory symptoms were as severe as before; and the abrasion of the epithelium had advanced downward across the center of the cornea. It formed a peculiar zig-zag line with short gray lines branching out alternately from each side at short intervals. Recognizing the significant character of this peculiar figure I at once gave the patient quinine On the evening of the same day the patient already experienced great relief; the pain subsided, the profuse secretion of tears ceased, and he could open and move the eye without the slightest discomfort.

I saw the patient again on February 5, when the eye was free from all irritation and redness; the epithelium of the cornea was restored, but in its center there was a small star-shaped opacity which greatly impaired the sight. This opacity gradually grew smaller, but it was still perceptible when I saw the patient a few months since.

II. M. Br., aged thirty-five, conductor on railroad, came under my care on January 20. One week ago he had an attack of fever, which was followed by severe pain in the right side of the head and in the right eye. He could not open the eye, because every attempt caused a sharp cutting pain, and ordinary day-light was intolerable. The tears were running down the cheek in a continuous current. The ocular conjunctiva was engorged, and there was decided pericorneal injection. In the cornea, near its upper margin, a small abrasion of the epithelium was all the lesion I could discover. I did not notice anything unusual about this abrasion at that time, and prescribed atropine ointment and a bandage. After one week the patient returned. He had suffered great pain all the time, but especially at night it was exceedingly severe. He had lost his appetite, and his tongue was coated. The abrasion in the cornea had increased in length so as to extend almost across the entire cornea; it exhibited, then, very distinctly Kipp's characteristic symptoms; and, therefore, quinine was given at once. On the evening of the same day already the pain had subsided, and the patient could sleep well. On the following day I observed a marked decrease of all the inflammatory symptoms. Three days later there was but a faint redness around the cornea ; no lachrymation or photophobia. The epithelium was repaired along the line of ulceration, but its peculiar figure was still plainly outlined by an opacity. In the course of six weeks this opacity was cleared up.

Inasmuch as these opacities are slow in clearing, and particularly tenacious in the central portion of the cornea, an early recognition of the nature of this corneal trouble is of paramount importance, because the appropriate treatment can arrest the ulcerative process before it encroaches upon the center of the cornea. This point will be well illustrated by the following case :

Mrs. B., aged thirty-six years, had intermittent fever at different times. On the evening of Feb. 15, while in a warm room, she had a chill, followed by high fever. Next day, soon after breakfast, her left eye became very red and painful, and the tears kept running down the cheek. It was an intense, sharp pain, aggravated by the slightest movement of the eyeball or upper portion of the lid, so that the lady thought it was caused by a foreign body under the eyelid. Instead of a foreign body, however, I found in the upper nasal section of the left cornea, very near its margin, a minute, slightly raised, gray spot, like a phlyctenule, from which a fine gray linear opacity proceeded downward. This gray line was only one millim. long, but the most valuable symptom in the case ; for at its lower end it branched out, thereby indicating the malarial keratitis in its incipient stage. I prescribed quinine (two grs. every two hours), and put a drop of atropine in the eye. After three doses of quinine were taken, the pain began to subside. The next day lachrymation, photophobia, pain and redness had disappeared, and two days later no trace of the corneal trouble could be discovered.

---

A PHYSICIAN of Erie, Pennsylvania, is training carrier pigeons for use in his practice. He leaves pigeons at places from which he wishes reports to be sent him.—*Boston Med. and Surg. Journal.*

